# Early and 3-Year Outcomes of Frozen Elephant Trunk Procedure with Evolving E-vita Hybrid Grafts: A Retrospective Single-Centre Cohort Study over 11 Years

**DOI:** 10.3390/jcdd12090368

**Published:** 2025-09-18

**Authors:** Isabelle Doll, Christoph Salewski, Luise Vöhringer, Michael Baumgaertner, Attila Nemeth, Christian Schlensak, Medhat Radwan

**Affiliations:** 1 Department of Thoracic and Cardiovascular Surgery, Tübingen University Hospital, 72076 Tübingen, Germany; christoph.salewski@med.uni-tuebingen.de (C.S.); attila.nemeth@med.uni-tuebingen.de (A.N.); christian.schlensak@med.uni-tuebingen.de (C.S.); medhat.radwan@med.uni-tuebingen.de (M.R.); 2 Department of Cardiovascular Surgery, Basel University Hospital, 4031 Basel, Switzerland; luise.voehringer@usb.ch

**Keywords:** frozen elephant trunk, aortic arch replacement, total arch replacement, aortic dissection, aortic aneurysm

## Abstract

Background/Objectives: The frozen elephant trunk (FET) technique is a cornerstone procedure for complex thoracic aortic pathologies. This single-center retrospective study evaluates early and midterm outcomes of total arch replacement (TAR) using three generations of the E-vita Open hybrid prosthesis over 11 years. Methods: From January 2013 to June 2024, 51 patients underwent TAR with the FET technique using the E-vita Open prostheses. Exclusion criteria were isolated ascending or descending aortic replacement, partial arch replacement, TAR without FET, and use of other stent grafts. We analyzed outcomes including in-hospital mortality, survival, stroke, spinal cord injury, and renal complications across three prosthesis generations. Results: The cohort included 52.9% males, with a mean age of 61.5 ± 10.51 years. FET as reoperation was performed in 52.9% patients. In-hospital mortality was 7.8% and, unexpectedly, only occurred with the newest E-vita Open Neo (23.5%), despite this being the latest generation. Overall survival was 72.5% at one year, 60.8% at two years, and 54.9% at three years. Stroke occurred in 17.6% with marked variation by pathology: 0% in dissection, 31.6% in aneurysm, and 13.6% in combined disease. Spinal cord injury occurred in 7.8%. Re-operation was a significant risk factor for complications and was strongly associated with renal complications (85.7% of dialysis patients) but not mortality. Secondary endovascular procedures were required in 49% of patients. Conclusions: The FET technique with E-vita Open prostheses demonstrates acceptable outcomes in high-risk patients with complex aortic pathologies. While perioperative morbidity is significant, particularly in reoperative cases, it varies significantly by underlying pathology and prosthesis generation, with unexpected trends suggesting that technological advancement does not automatically translate into improved outcomes. Despite this, the procedure enables comprehensive management of extensive aortic disease through a staged approach.

## 1. Introduction

The surgical treatment of extensive aortic arch and descending thoracic aortic pathologies has shifted over the past two decades from multi-staged repairs to hybrid techniques [1,2]. Among these, the Frozen Elephant Trunk (FET) has become a key strategy, combining open arch replacement with endovascular stent grafting in a single stage [3,4]. This approach enables comprehensive treatment of the arch and proximal descending aorta, creates a stable landing zone for future endovascular interventions, and involves improved long-term management of complex thoracic aortic disease. Meta-analyses suggest that while conventional repair may carry lower stroke risk, FET achieves superior completeness of repair [5,6,7].

Clinical outcomes support the efficacy of FET. Five-year survival rates after FET have been reported as high as 95%, compared to approximately 70% after conventional arch replacement [8]. Although the risk of spinal cord injury is slightly higher with FET, the long-term therapeutic possibilities outweigh the risks [9,10,11]. Registry analyses further demonstrate the durability of the procedure, with the International E-vita Open Registry collecting data on more than 1200 patients across 19 centers since 2008 [12].

The evolution of dedicated hybrid prostheses has facilitated the reproducibility of the technique. The E-vita Open system (JOTEC/Artivion), introduced in 2005, was the first commercially available hybrid graft in Europe and has undergone iterative refinements, culminating in the E-vita Open Neo [13,14,15,16]. These developments have expanded surgical flexibility and broadened the indications of FET to include chronic thoracic aortic dissection, complex type B dissections not amenable to endovascular repair, thoracic aneurysms, and selected acute type A dissections with arch involvement or distal malperfusion [17,18].

Despite encouraging data, multicenter studies often remain heterogeneous in patient selection, surgical techniques, and perioperative management. This limits their applicability to daily practice. To address this, we conducted a single-center analysis of patients treated exclusively with the E-vita prostheses under standardized peri- and intraoperative protocols. We focus on transfusion requirements and neurological complications as clinically relevant outcomes in this high-risk population.

## 2. Materials and Methods

### 2.1. Ethical Statement

Approval for this retrospective study was obtained from the local institutional review board and ethics committee of the University of Tübingen (Project number 005/2025BO2) on 15 January 2025. Due to the retrospective character of this study, written consent was waived.

### 2.2. Study Design and Patient Population

This retrospective study was conducted at the University Hospital Tübingen, Germany. Between January 2013 and June 2024, all patients who underwent thoracic aortic surgery were identified from our institutional Aortic Registry. A total of 51 patients who underwent total aortic arch replacement (TAR) using the FET technique with an E-vita Open stent graft were included. It is important to note that the distribution of prosthesis generations reflects the chronological evolution of our institutional practice, with outcomes potentially influenced by concurrent improvements in surgical experience and perioperative management rather than device characteristics alone.

Exclusion criteria comprised isolated ascending or descending aortic replacement, partial arch replacement, TAR without FET, and the use of non-E-vita stent grafts. All procedures were performed by experienced cardiac surgeons and included both elective and emergency operations.

The decision to perform FET rather than conventional total arch replacement or alternative hybrid approaches was based on several institutional criteria developed over the study period:

Anatomical Criteria:

- Extensive aortic disease involving both the arch and proximal descending aorta (>5 cm from the left subclavian artery);
- Chronic dissection with false lumen patency extending into the descending aorta;
- Aneurysmal disease with diameter > 4.5 cm in the proximal descending aorta;
- Unsuitable anatomy for pure endovascular approaches due to inadequate landing zones.

Clinical Criteria:

- Reoperative cases where conventional approaches would be technically challenging;
- Younger patients (<70 years) requiring long-term durability;
- Emergency presentations requiring immediate definitive treatment;
- Planned staged approach with anticipated future endovascular interventions.

Contraindications to FET:

- Severe peripheral vascular disease precluding femoral access for stent graft deployment;
- Extensive calcification of the descending aorta;
- Previous extensive descending aortic surgery;
- Prohibitive operative risk for prolonged procedures.

### 2.3. Analyzed Parameters

Comprehensive perioperative data were retrospectively extracted from the institutional clinical information system.

Preoperative variables included demographic characteristics, urgency of surgery (elective, urgent, emergent), type of intervention (primary vs. redo surgery), comorbidities, and relevant laboratory values. Operative risk assessment was performed using the EuroSCORE II calculator.

Intraoperative data comprised total operative time, duration of cardiopulmonary bypass (CPB), aortic cross-clamp time, and durations of selective cerebral perfusion and reperfusion, as well as details of any concomitant procedures. The type of E-vita prosthesis used, the cerebral perfusion strategy (unilateral, bilateral, trilateral), and the Ishimaru zone of distal anastomosis were recorded. Additionally, intraoperative nadir body temperature, peak lactate levels, and transfusion requirements for blood products and hemostatic agents were documented.

### 2.4. Preoperative Management

All elective surgery patients underwent preoperative pulmonary function tests, laboratory analysis, computed tomography (CT) and echocardiography, while in emergency patients, only CT scans were performed.

To reduce the risk of spinal cord ischemia, all elective patients in our institution underwent preoperative cerebrospinal fluid (CSF) drainage targeting a CSF pressure < 10 mmHg (drainage volume ≤ 10 mL/h).

To facilitate intraoperative trilateral antegrade perfusion, patients underwent a Carotid-subclavian (CS) bypass 1–2 days before the day of FET surgery, when applicable. The primary indication for a preoperative CS bypass was to facilitate a trilateral antegrade cerebral perfusion strategy during the FET procedure when the Circle of Willis was not well developed. In our early experience, we encountered challenges in achieving adequate perfusion of the left subclavian and left carotid artery territory in cases with complex arch anatomy or significant atherosclerotic disease. The decision to perform a preoperative bypass was made on a case-by-case basis, considering the individual patient’s anatomy, the extent of the planned aortic resection, the adequacy of the Circle of Willis, and the perceived risk of spinal cord ischemia.

### 2.5. Surgical Technique

All operations were performed via median sternotomy under general anesthesia. After systemic heparinization, CPB was established via cannulation of the right axillary artery using an 8 mm Dacron Prosthesis (for antegrade cerebral perfusion) and bicaval venous drainage. Left heart decompression was achieved through a left ventricular vent via the right superior pulmonary vein.

Core cooling was applied to a bladder temperature of 25–28 °C. Myocardial protection was achieved using intermittent cold blood cardioplegia.

Subsequently, circulatory arrest was initiated, and antegrade selective cerebral perfusion (ASCP) was commenced. Cerebral oxygen saturation was monitored with bifrontal cerebral Near-Infrared Spectroscopy (NIRS; Medtronic/Covidien INVOS™, Minneapolis, MN, USA and Masimo O3^®^ Regional oximetry, Irvine, CA, USA). Depending on individual anatomy, NIRS decline, and surgeon preference, unilateral (right axillary artery), bilateral (right axillary and left carotid artery), or trilateral (right axillary, left carotid, and left axillary artery) ASCP was employed.

The aortic arch was resected, and the true lumen of the descending thoracic aorta was carefully identified. The E-vita prosthesis (E-vita Open, Open Plus, or Open Neo), selected based on preoperative imaging, was deployed into the descending aorta under direct vision with the aid of a guiding wire from the femoral artery and secured via a distal anastomosis using a continuous polypropylene suture. Care was taken to achieve a tension-free and hemostatic anastomosis.

Following prosthesis deployment, systemic perfusion was gradually restored via a side branch of the vascular graft when using the new Evita Open Neo stent generation. The supra-aortic vessels were then sequentially reimplanted onto the branches of the prosthesis in an island or individual fashion, depending on anatomic considerations. Finally, proximal anastomosis to the ascending aorta or a separate graft (in cases of additional root or ascending aortic repair) was performed.

Concomitant procedures (e.g., valve replacement, coronary artery bypass grafting) were performed as indicated.

Rewarming was initiated, and the heart was de-aired and reperfused. Hemostasis was secured, and the patient was weaned from CPB in standard fashion. Hemodynamic targets during reperfusion included a mean arterial pressure ≥ 70 mmHg.

Afterwards, patients were transferred to the Intensive Care Unit (ICU) for further monitoring, including standardized neurologic assessments and daily serum creatinine monitoring.

A video demonstrating the implantation of the E-vita Open Neo prosthesis is provided in the Supplementary Material (Video S1).

### 2.6. Study Endpoints and Follow-Up

The primary endpoint was all-cause in-hospital mortality. Secondary endpoints included perioperative stroke, spinal cord injury, reoperation for bleeding, acute kidney injury requiring renal replacement therapy, and other major complications. Midterm outcomes encompassed survival at one, two, and three years and the need for secondary thoracic endovascular aortic repair (TEVAR) or branched endovascular aortic repair (BEVAR).

Stroke was defined as a clinically apparent neurological deficit confirmed by imaging as either ischemic or hemorrhagic lesion. Spinal cord injury was diagnosed based on clinical presentation and confirmed using magnetic resonance imaging (MRI).

Survival and reintervention data were obtained from outpatient visits and telephone interviews. Imaging follow-up with contrast-enhanced CT scans was scheduled at discharge, six months, and annually thereafter.

Fourteen patients were operated on during the last three years of the study period and therefore did not complete a full three-year follow-up, which must be considered when interpreting the reported survival rate. All patients, however, had at least one year of follow-up. Owing to the retrospective study design, it was not always possible to distinguish between death and loss to follow-up.

### 2.7. Statistical Analysis

Statistical analyses were performed using SPSS version 30.0 (IBM Corp., Armonk, NY, USA). Normality of data distribution was assessed using the Shapiro–Wilk test. Continuous variables are presented as mean ± standard deviation (SD) if normally distributed; otherwise, they are reported as median and interquartile range (IQR). Categorical variables are expressed as absolute numbers and percentages. Due to the descriptive nature of this study and limited sample sizes within prosthesis generation subgroups, formal comparative statistical analyses were not performed. Subgroup analyses are presented descriptively where sample sizes permit meaningful interpretation.

## 3. Results

A total of 51 patients underwent TAR using the FET technique with various generations of the E-vita Open hybrid prosthesis between January 2013 and June 2024 in our center.

The study cohort had a mean age of 61.5 ± 10.51 years, and 27 patients (52.9%) were male. Notably, over half of the cohort (n = 27, 52.9%) underwent the procedure as a reoperation. Among these reoperation cases, 15 patients (29.4%) had a history of prior acute type A dissection repair, 5 (9.8%) had previous ascending aortic replacement, and 7 (13.7%) had undergone other cardiac procedures.

Regarding comorbidities, arterial hypertension was present in the majority of patients (90.2%), and 56.9% had hyperlipidemia. A history of smoking was reported in 33.3%, while 5.9% had a diagnosis of chronic obstructive pulmonary disease (COPD). Previous stroke was documented in 5 patients (9.8%).

Preoperative renal function varied across the cohort, with 13 patients (25.5%) showing no renal impairment and the majority presenting with mild to moderate dysfunction: 4 patients (7.8%) had a Glomerular Filtration Rate (GFR) > 89 mL/min (Stage 1), 23 (45.1%) had a GFR of 60–89 mL/min (Stage 2), and 10 (19.6%) had a GFR of 30–59 mL/min (Stage 3). Severe renal insufficiency was rare, with only one patient (2.0%) in Stage 4 (GFR 15–29 mL/min) and none in Stage 5. No patient required dialysis preoperatively. Diabetes mellitus was present in 11 patients (21.5%), of whom 4 (7.8%) were insulin-dependent, 3 (5.9%) were treated with oral antidiabetic drugs (OAD), and 4 (7.8%) were unmedicated.

Patients were classified by underlying pathology into three main diagnostic categories: aortic dissection (n = 10, 19.6%), thoracic aortic aneurysm without dissection (n = 19, 37.3%), and a combination of both (n = 22, 43.1%).

80.4% of procedures were performed electively, 13.7% as urgent, and 5.9% in an emergency situation. The Median EuroSCORE II was 3.65 (1.85–5.45), indicating a moderate preoperative surgical risk profile.

A CS-bypass before FET implantation was performed in 16 patients (31.4%).

Patient characteristics are shown in Table 1.

All patients received one of three generations of the E-vita prosthesis: the original E-vita Open (n = 23, 45.1%), the E-vita Open Plus (n = 11, 21.6%), or the most recent E-vita Open Neo (n = 17, 33.3%). Preoperative CSF drainage was employed in 76.5% of patients (n = 39), in accordance with our institutional protocols aimed at reducing the risk of spinal cord injury.

The mean CPB-time was 241.67 ± 55.93 min, with aortic cross-clamp and selective cerebral perfusion times averaging 114.06 ± 39.55 min and 76.85 ± 29.91 min, respectively. Antegrade cerebral perfusion was administered unilaterally via the right axillary artery in 36 patients (70.6%), bilaterally in 7 patients (13.7%), and trilaterally (including left subclavian artery perfusion) in 8 patients (15.7%). Circulatory arrest was employed in 30 patients (58.8%), with a mean arrest duration of 74.2 ± 33.7 min. The distal anastomosis was performed in Ishimaru zone 0 in 14 patients (27.5%), zone 2 in another 14 (27.5%), and zone 3 in 17 (33.3%).

Reimplantation of the arch vessels included all three (brachiocephalic trunk, left common carotid artery, and left subclavian artery) in 24 cases (47.1%) and only the trunk and the carotid artery in 26 cases (51.0%). Concomitant cardiac procedures, such as aortic valve replacement or coronary artery bypass grafting, were performed in 10 patients (19.6%).

The maximum intraoperative lactate level was a median of 6.8 mmol/L (4.6–8.6).

Intraoperative parameters are displayed in Table 2.

Intraoperative transfusion requirements are summarized in Table 3. Red blood cell (RBC) transfusion was administered to 45 patients, with a median volume of 1500 mL (900–2550 mL). Platelet concentrates were given to 44 patients, with a median of 900 mL (600–1612.5 mL). Fresh frozen plasma (FFP) was administered to 29 patients, with a median volume of 1500 mL (900–3000 mL).

Prothrombin complex concentrate (PCC) was used in 47 patients, with a median dose of 5000 I.U. (4000–8000 I.U.). Fibrinogen was administered to 46 patients, with a median of 6 g (4–8 g).

In selected cases, specific factor concentrates were required: recombinant activated factor VII (NovoSeven) was used in 7 patients with a median dose of 8 mg (6.3–12 mg), Haemate was administered to 13 patients with a median of 3000 I.U. (2000–4500 I.U.), and Fibrogammin was used in 7 patients, with a median dose of 1250 I.U. (1250–2500 I.U.).

Table 4 shows postoperative cohort characteristics and complications. The median length of stay in the ICU was 6 days (4–11 days), with a median total hospitalization duration of 20 days (16–30 days).

The in-hospital mortality rate was 7.8% (n = 4). Causes of death included multiple organ failure in two patients (3.9%), stroke in one patient (2.0%), and low cardiac output syndrome in another (2.0%). Neurologic complications were not uncommon; 9 patients (17.6%) suffered perioperative strokes, and 4 patients (7.8%) developed spinal cord injury resulting in persistent neurological deficits at discharge. Re-exploration for postoperative bleeding was required in 10 patients (19.6%), while delirium was documented in 11 patients (21.6%).

Postoperative renal function was impaired in a significant proportion of patients. Only 2 patients (3.9%) had no signs of renal insufficiency, and another 2 (3.9%) had a GFR > 89 mL/min (Stage 1). Mild to moderate dysfunction was observed in 8 (15.7%) and 15 patients (29.4%) in Stages 2 and 3, respectively. Advanced renal impairment was present in 7 patients (13.7%) with Stage 4 and 3 patients (5.9%) with Stage 5 renal failure. Notably, 14 patients (27.5%) required postoperative dialysis.

During the follow-up period, 25 patients (49%) required secondary endovascular interventions, including TEVAR or BEVAR, primarily for distal aortic progression or as part of a planned staged approach. Survival analysis showed a one-year overall survival of 72.5% (n = 37), declining to 60.8% (n = 31) at two years and 54.9% (n = 28) at three years.

Since only 37 patients completed the full three-year follow-up, the adjusted three-year survival rate was 75.7% (28 of 37 patients).

As underlying pathology and used E-vita prosthesis type may significantly influence postoperative outcomes, results are stratified by pathology in Table 5 and by prosthesis type in Table 6.

When stratified by underlying pathology, in-hospital mortality was comparable across groups, ranging from 4.5% in the combined group to 10.0–10.5% in dissection and aneurysm patients. Median EuroSCORE II was lowest in patients with dissection and highest in those with combined pathology. Neurological complications showed relevant differences: no strokes occurred in the dissection group, whereas 31.6% of aneurysm patients and 13.6% of patients with combined pathology were affected. Rates of paraplegia were similar across groups (5–10%). Renal replacement therapy was markedly more frequent in aneurysm (31.6%) and combined patients (31.8%) than in dissections (10%). Operative duration was longest in combined pathology, accompanied by prolonged cerebral perfusion times.

Outcomes differed markedly between prosthesis generations. In-hospital mortality was absent in patients treated with the E-vita Open and Open Plus, whereas it reached 23.5% with the E-vita Open Neo. Neurological complications showed distinct patterns. Stroke occurred in about one fifth of patients treated with the Open and Neo grafts, whereas none were observed in the Open Plus group. Paraplegia was rare overall, though slightly more frequent with newer prostheses. Postoperative dialysis was required most often with the Open Neo (41.2%), compared to ~20% with the other grafts. Three-year survival was most favorable with the Open Plus (81.8%), intermediate with the Open (65.2%), and lowest with the Open Neo (23.5%).

Given the predominance of neurological and renal complications, detailed risk factor analyses are presented in Table 7 and Table 8.

Patients with stroke did not differ significantly in age, CPB, or cross-clamp times compared to those without stroke. However, cerebral perfusion times were longer in the stroke group (90 vs. 74 min). Re-operations were more frequent among stroke patients (66.7% vs. 50.0%). Prosthesis type showed a notable distribution: strokes occurred predominantly with the E-vita Open (55.6%) and Open Neo (44.4%), but not with the Open Plus. Perfusion strategy and circulatory arrest times were comparable between groups. Maximum lactate values tended to be higher in stroke patients.

Patients requiring postoperative dialysis (n = 14) were slightly older and more often re-operated (85.7% vs. 40.5%). Preoperative renal dysfunction (GFR < 60 mL/min) was also more frequent in this group (28.6% vs. 10.8%). Intraoperatively, CPB and cross-clamp times were similar, but dialysis patients required higher transfusion volumes, particularly FFP and PCC.

Neurological complications were more common in the dialysis group, with stroke occurring in 35.7% compared to 10.8% in patients without dialysis. Re-sternotomy was also more frequent (35.7% vs. 13.5%). Length of stay in ICU and total hospitalization were markedly prolonged in dialysis patients (median 21 vs. 5 days in ICU; 35 vs. 18.5 days until discharge). In-hospital mortality was higher in the dialysis group (14.3% vs. 5.4%), though absolute numbers were small.

In the surgical context, neurologic and renal complications occurred more frequently in patients undergoing reoperative procedures. Stroke was more prevalent in reoperations (n = 6, 66.7%) compared to primary surgeries (n = 3, 33.3%). Similarly, three out of four cases of spinal cord injury (75.0%) were observed in the reoperative subgroup. The need for postoperative dialysis and re-sternotomy was also markedly higher in reoperations, affecting 85.7% (12/14) and 60.0% (6/10) of these patients, respectively.

## 4. Discussion

This single-center retrospective study reports early and three-year outcomes of FET repair with three successive E-vita Open graft generations in high-risk patients undergoing either primary or redo surgery. Early results were encouraging: 30-day mortality was 7.8%, and overall survival reached 72.5%, 60.7%, and 54.9% at one, two, and three years, respectively. These outcomes reinforce the accumulating evidence that FET is both safe and effective for extensive aortic disease. However, the distribution of prosthesis generations over our study period introduces potential confounding, as outcomes may reflect not only device characteristics but also evolving surgical expertise, patient selection criteria, and perioperative management protocols. This limitation must be considered when interpreting prosthesis-specific outcomes.

Comparison with the existing literature reveals that our observed in-hospital mortality rate of 7.8% is within the range reported by other studies and registries. For example, the 19-center International E-vita Open Registry noted a 12% 30-day mortality [19]. A systematic review quotes 30-day mortality between 5% and 14% [20]. The slightly lower mortality observed in our cohort could be attributed to several factors, including evolving surgical techniques, improved patient selection, and advancements in perioperative management over the 11-year study period. The continuous refinement of the E-vita Open prosthesis, from the original E-vita Open to the E-vita Open Plus and E-vita Open Neo, likely contributed to these improved outcomes by offering enhanced flexibility and reduced procedural complexity [21,22]. However, our unexpected finding that all in-hospital mortality occurred with the newest E-vita Open Neo (23.5% mortality rate) challenges assumptions about linear technological improvement. This paradoxical result likely reflects increased case complexity, learning curve effects with new technology, and selection bias rather than device inferiority. The E-vita Open Neo was introduced during a period when our institutional experience allowed us to tackle more challenging cases, potentially explaining these outcomes.

In relation to conventional TAR treatment approaches, our observed in-hospital mortality rate of 7.8% compares favorably with reported conventional TAR mortality rates in similar high-risk populations [23]. The Society of Thoracic Surgeons database reports conventional TAR mortality rates ranging from 8–15% in comparable patient populations, with higher rates observed in reoperative cases [24].

The key advantage of FET over conventional TAR lies not only in comparable early mortality but in the comprehensive nature of the repair. While conventional TAR addresses only the arch, leaving descending aortic pathology untreated and requiring subsequent procedures, FET provides definitive treatment of both arch and proximal descending aortic disease in a single operative session [5,25].

The completeness of repair achieved with FET is particularly evident in our cohort, where 43.1% of patients had combined dissection and aneurysmal disease extending into the descending aorta. Conventional TAR in these patients would have left significant residual pathology, necessitating subsequent interventions with their associated risks and costs. The FET approach eliminated this staged approach, providing immediate definitive treatment of the entire pathological segment [8].

Debranching procedures with subsequent TEVAR represent an alternative hybrid approach to extensive arch disease [10]. While debranching may have lower immediate neurological complications in some series, it requires multiple complex anastomoses and has limited applicability in emergency settings [11].

Our institutional experience suggests that FET offers several advantages over debranching approaches like a simplified surgical technique with fewer anastomoses, maintained anatomical relationships of arch vessels, and superior applicability in emergency situations where time is critical.

Furthermore, the long-term durability of debranching procedures remains a concern, particularly regarding the patency of bypass grafts and the integrity of multiple anastomoses [26].

Pure endovascular approaches, including zone 0 TEVAR with branched devices, are limited by several factors that make FET more applicable. Zone 0 TEVAR is constrained by anatomical requirements including adequate landing zones, appropriate angulation, and absence of significant calcification. Long-term concerns about proximal seal durability and the complexity of branched devices further limit the applicability [27].

Peri-operative morbidity remained substantial: stroke at 17.6%, spinal cord injury at 7.8%, re-exploration for bleeding at 19.6%, and dialysis-requiring kidney injury at 27.5%. Although high, these rates are consonant with the extreme risk profile of the cohort and the procedure’s complexity. For example, reported rates of stroke in FET series typically range from 5.4% to 9.3% and for spinal cord injury from 2% to 8.9% [20].

Our stroke rate, while higher than some reported series, reflects the complexity of our patient population and provides important insights into modifiable risk factors [28]. The most significant finding in our analysis is that 66.7% of strokes occurred in reoperative cases, despite reoperations comprising only 52.9% of the cohort. This disproportionate representation suggests that prior cardiac surgery significantly increases neurological risk, likely due to altered anatomy, adhesions, and prolonged operative times. The presence of scar tissue and altered vascular anatomy in reoperative cases may compromise cerebral perfusion strategies and increase embolic risk. Our detailed analysis revealed stroke rates of 77.8% (n = 7) with unilateral perfusion and 11.1% (n = 1) with bilateral and trilateral perfusion, respectively. While the numbers are too small to draw definitive conclusions, there does not appear to be a clear protective effect of more extensive cerebral perfusion strategies. The variation may reflect selection bias, as bilateral perfusion was often employed in more complex cases with higher baseline neurological risk.

The most dramatic finding was the variation in stroke risk by underlying pathology: 0% in pure dissection, 31.6% in aneurysm, and 13.6% in combined pathology patients. This substantial difference likely reflects varying atherosclerotic burden and embolic risk profiles. Aneurysmal disease is often associated with severe atherosclerosis, increasing embolic potential during manipulation. In contrast, dissection patients are typically younger with less atherosclerotic burden, potentially explaining their remarkably low stroke rate despite the complexity of their aortic pathology.

Also, technical factors like the choice of arterial cannulation site significantly impacts stroke risk in FET procedures. Our institutional preference for axillary artery cannulation in most cases reflects current best practices for cerebral protection, though cannulation site complications and technical challenges in reoperative cases may contribute to embolic events [21].

Furthermore, extended periods of hypothermic circulatory arrest, particularly in complex reoperative cases, may increase the risk of neurological complications [29]. Our mean circulatory arrest time of 74.20 ± 33.66 min still falls within acceptable ranges although slightly longer than reported in contemporary series, though individual cases, especially stroke patients, with prolonged arrest times may have contributed to adverse neurological outcomes.

The manipulation of diseased aortic tissue during FET deployment, particularly in cases with extensive atherosclerotic disease or previous surgical interventions, may increase the risk of cerebral embolism [30]. Careful surgical technique and appropriate use of cerebral protection strategies are essential for minimizing these risks.

Regarding postoperative paraplegia, the length of the aorta covered by the stent graft component directly correlates with spinal cord injury risk [31]. Our institutional approach of tailoring the stent graft length to individual anatomy while ensuring adequate coverage of pathological segments as well as staged approaches represent a balance between therapeutic efficacy and neurological safety. The identification and preservation of critical intercostal arteries, when technically feasible, remain an important consideration in FET planning. Perioperative blood pressure management and the use of cerebrospinal fluid drainage in high-risk patients represent important strategies for spinal cord protection [32]. Our institutional protocols for spinal cord protection have evolved over the study period, potentially contributing to improved outcomes in more recent cases.

The high rate of acute kidney injury requiring dialysis in our series (27.5%) exceeds rates reported in some contemporary FET series, which typically range from 10–20% [20]. This difference may reflect our institutional threshold for initiating renal replacement therapy, the complexity of our patient population, or differences in perioperative management protocols. The predominance of renal failure in re-operative cases (85.7% of dialysis patients) underscores the particular vulnerability of this patient subgroup and represents one of the strongest risk associations in our series. Multiple pathophysiologic factors likely contribute to this elevated risk, including more pronounced systemic inflammatory responses in re-operations, longer operative times with extended cardiopulmonary bypass, technical challenges leading to prolonged ischemic times, massive transfusion-related complications and coagulopathy, and shared risk factors between neurological and renal complications. The association between dialysis requirements and concurrent stroke (35.7% of dialysis patients had strokes) suggests common pathophysiologic mechanisms including embolic phenomena and hypoperfusion.

The cohort demonstrated substantial intraoperative blood loss, necessitating the transfusion of red blood cells, platelets, fresh frozen plasma, and various coagulation factors. These findings align with previous reports emphasizing the high hemorrhagic risk during extensive aortic repair due to prolonged CPB, hypothermia, and inherent coagulopathy. The frequent use of PCC and fibrinogen reflects current hemostatic management strategies. In selected cases, more potent agents such as recombinant factor VII (Novoseven), Haemate, or Fibrogammin were required. Notably, transfusion requirements and coagulation management are seldom the primary focus of studies on the frozen elephant trunk technique, resulting in a limited evidence base for guiding perioperative therapy [22,33].

The long-term survival rates in our study (72.5% at one year, 60.7% at two years, and 54.9% at three years) demonstrate the durability of the FET technique in managing complex aortic diseases. These figures are comparable to, and in some cases exceed, those reported in other long-term follow-up studies. For instance, a single center’s 15-year experience with FET using different hybrid grafts reported overall survival rates of approximately 73% at one year, 70% at two years, and 64% at five years [34].

It should be noted that not all patients have completed the full three-year follow-up. As this mainly concerns the most recent cases, particularly in the E-vita Open Neo group, the incomplete follow-up may contribute to the lower survival rate observed in this subgroup. The sustained survival beyond the immediate postoperative period underscores the effectiveness of the FET procedure in providing a definitive solution for extensive aortic pathologies, preventing further progression of the disease, and improving patient prognosis.

The unexpected observation that re-operation was not associated with higher in-hospital mortality (3.7% vs. 12.5% in primary procedures) contradicts conventional expectations for reoperative cardiac surgery. This finding may reflect several factors like improved patient selection in re-operative cases, where only patients deemed suitable for additional surgical risk were offered the procedure; enhanced surgical experience with complex cases over time; better perioperative management protocols developed through institutional learning; and statistical variation due to small sample size. However, the significantly higher morbidity in re-operative cases (particularly renal failure and neurological complications) suggests that while immediate survival may be preserved, the physiological burden remains substantial.

Regarding the high rate of secondary interventions (49%), while this may initially appear concerning, it reflects the conceptual strength of the FET strategy as a platform for staged management of extensive aortic disease. However, we acknowledge the significant clinical burden this represents, including inherent procedural risks with each reintervention, additional hospitalizations and patient psychological burden, lifelong surveillance requirements with serial imaging studies, cumulative healthcare costs and resource utilization, and the potential need for multiple reinterventions over a patient’s lifetime. A multidisciplinary team approach is essential for optimal patient selection and long-term management planning.

The evolution of the E-vita Open prosthesis, particularly the E-vita Open Neo, has introduced enhanced flexibility into surgical approaches, allowing for patient-tailored interventions across different arch zones [21]. This adaptability is crucial for managing the diverse anatomical presentations of complex aortic diseases. The continuous innovation in hybrid graft technology aims to simplify surgical procedures, reduce operative times, and ultimately improve patient outcomes [22]. Our study, spanning the use of three generations of the E-vita Open prosthesis, indirectly supports the positive impact of these technological advancements on clinical results.

## 5. Limitations

This retrospective single-center study has several important limitations that must be acknowledged. The descriptive nature of our analysis, while providing valuable institutional experience, does not include formal comparative statistical analyses between prosthesis generations or with alternative treatment approaches.

We did not perform propensity score matching or multivariable adjustments to account for differences between patient groups, and the small sample size (n = 51) distributed across three prosthesis generations limits our ability to draw definitive conclusions about differences between groups. The retrospective design may introduce selection bias related to institutional referral patterns, surgeon preferences, and evolving patient selection criteria over the 11-year study period.

Our findings may have limited generalizability as they reflect the experience of a single center and may not be applicable to other institutions with different patient populations, surgical techniques, or perioperative management protocols.

The use of different prosthesis generations across different time periods introduces potential confounding from evolving surgical techniques, perioperative management protocols, and institutional learning curves. Improvements in outcomes over time may reflect not only prosthesis evolution but also accumulated institutional experience. The unexpected adverse outcomes with the newest prosthesis generation (E-vita Open Neo) exemplify this confounding, as they likely reflect increased case complexity and changing patient selection rather than device performance. Due to the limited and unbalanced sample sizes for each prosthesis, formal statistical comparisons between prosthesis generations were not feasible.

Long-term survival data may be influenced by incomplete follow-up, as some patients were lost to follow-up during the observation period, and not all patients were eligible for full three-year follow-up since procedures performed within the last year prior to data collection were included. Our study does not include concurrent control groups receiving conventional total arch replacement or alternative hybrid approaches, limiting our ability to make definitive statements about the relative advantages of FET.

## 6. Conclusions

This retrospective single-center analysis of 51 patients undergoing FET procedures with three generations of E-vita Open prostheses demonstrates that FET offers a viable and effective treatment option for high-risk patients, including those undergoing reoperative procedures, with acceptable early and midterm mortality rates despite significant perioperative morbidity. Our unexpected finding that newer prosthesis generations did not demonstrate improved outcomes serves as an important reminder that technological advancement does not automatically translate into clinical improvement, particularly when patient complexity and practice patterns evolve concurrently. The E-vita Open prostheses also facilitate long-term management through subsequent endovascular interventions. Our experience demonstrates that with appropriate patient selection, meticulous surgical technique, and comprehensive perioperative care, FET can achieve acceptable outcomes even in high-risk populations.

## Figures and Tables

**Table 1:** Baseline characteristics.

Variable	Total Cohort (n = 51)
Age (years)	61.55 ± 10.51
Gender (male)	27 (52.9%)
Height (cm)	170.00 (163.0–176.0)
Weight (kg)	82.00 (71.0–96.0)
Diabetes OAD Insulin-dependent Unmedicated	3 (5.9%) 4 (7.8%) 4 (7.8%)
Hypertension	46 (90.2%)
Hyperlipidemia	29 (56.9%)
COPD	3 (5.9%)
Smoking history	17 (33.3%)
Stroke	5 (9.8%)
Renal insufficiency 0: none 1: GFR > 89 (mL/min) 2: GFR 60–89 (mL/min) 3: GFR 30–59 (mL/min) 4: GFR 15–29 (mL/min) 5: GFR < 15 (mL/min) Dialysis	13 (25.5%) 4 (7.8%) 23 (45.1%) 10 (19.6%) 0 (0.0%) 1 (2.0%) 0 (0.0%)
EuroSCORE II	3.65 (1.85–5.45)
Aortic pathology Dissection Aneurysm Combination	10 (19.6%) 19 (37.3%) 22 (43.1%)
Type of surgery Elective Urgent Emergent	41 (80.4%) 7 (13.7%) 3 (5.9%)
CS-Bypass before FET	16 (31.4%)
Previous Cardiac Surgery Type A Dissection Ascendens Replacement Other	27 (52.9%) 15 (29.4%) 5 (9.8%) 7 (13.7%)

Values shown as mean (±SD), median (IQR), or n (%). OAD: Oral Antidiabetic Drugs; COPD: Chronic Obstructive Pulmonary Disease; GFR: Glomerular Filtration Rate; CS-Bypass: Carotid-Subclavian Bypass; FET: Frozen Elephant Trunk.

**Table 2:** Intraoperative parameters.

Variable	Total Cohort (n = 51)
CSF drainage	39 (76.5%)
Duration of surgery (min)	480.36 ± 126.43
CPB-time (min)	241.67 ± 55.93
Cross-clamp-time (min)	114.06 ± 39.55
Reperfusion (min)	79.16 ± 32.84
Circulatory arrest (min)	74.20 ± 33.66 (n = 30)
Cerebral perfusion (min)	76.85 ± 29.91
Lowest temperature (°C)	27.9 (26.0–28.0)
Perfusion strategy Unilateral Bilateral Trilateral	36 (70.6%) 7 (13.7%) 8 (15.7%)
Prosthesis type Open Open Plus Open Neo	23 (45.1%) 11 (21.6%) 17 (33.3%)
Ishimaru zone of distal anastomosis 0 2 3	14 (27.5%) 14 (27.5%) 17 (33.3%)
Arch vessel reimplantation Truncus, carotid, subclavia Truncus, carotid	24 (47.1%) 26 (51.0%)
Concomitant procedure ACB Valve Both ASD-closure	5 (9.8%) 3 5.9%) 1 (2.0%) 1 (2.0%)
Max. Lactate (mmol/L)	6.8 (4.6–8.6)

Values shown as mean (±SD), median (IQR), or n (%). CSF: Cerebrospinal Fluid; CPB: Cardiopulmonary Bypass; ACB: Aorto-Coronary Bypass; ASD: Atrial Septal Defect.

**Table 3:** Intraoperative transfusion of blood and coagulatory components.

Variable	Total Cohort (n = 51)
RBCs (mL)	1500 (900–2550) (n = 45)
Platelets (mL)	900 (600–1612.5) (n = 44)
FFP (mL)	1500 (900–3000) (n = 29)
PCC (I.U.)	5000 (4000–8000) (n = 47)
Fibrinogen (g)	6 (4–8) (n = 46)
NovoSeven (mg)	8 (6.3–12) (n = 7)
Haemate (I.U.)	3000 (2000–4500) (n = 13)
Fibrogammin (I.U.)	1250 (1250–2500) (n = 7)

Values shown as median (IQR). RBCs: Red Blood Cells; FFP: Fresh Frozen Plasma; PCC: Prothrombin Complex Concentrate.

**Table 4:** Postoperative cohort characteristics and complications.

Variable	Total Cohort (n = 51)
LOS ICU (d)	6 (4–11)
Time to discharge (d)	20 (16–30)
Re-sternotomy	10 (19.6%)
Stroke	9 (17.6%)
Renal insufficiency 0: none 1: GFR > 89 (mL/min) 2: GFR 60–89 (mL/min) 3: GFR 30–59 (mL/min) 4: GFR 15–29 (mL/min) 5: GFR < 15 (mL/min) Dialysis	2 (3.9%) 2 (3.9%) 8 (15.7%) 15 (29.4%) 7 (13.7%) 3 (5.9%) 14 (27.5%)
Delirium	11 (21.6%)
Multiorgan failure	4 (7.8%)
Paraplegia	4 (7.8%)
Survival 30 d 1 y 2 y 3 y	47 (92.2%) 37 (72.5%) 31 (60.8%) 28 (54.9%)
In-hospital mortality	4 (7.8%)
In-hospital mortality in re-operations	1 (3.7%)
Subsequent endovascular procedure (TEVAR/BEVAR)	25 (49.0%)

Values shown as median (IQR) or n (%). LOS: Length of Stay; ICU: Intensive Care Unit; GFR: Glomerular Filtration Rate; TEVAR: Thoracic Endovascular Aortic Repair; BEVAR: Branched Endovascular Aortic Repair.

**Table 5:** Outcomes by Underlying Pathology.

Variable	Dissection (n = 10)	Aneurysm (n = 19)	Combined (n = 22)
In-hospital mortality	1 (10.0%)	2 (10.5%)	1 (4.5%)
EuroSCORE II	2.14 (1.85–3.55)	3.65 (1.75–5.98)	3.91 (2.21–6.59)
Stroke	0 (0.0%)	6 (31.6%)	3 (13.6%)
Paraplegia	1 (10.0%)	1 (5.3%)	2 (9.1%)
Postoperative dialysis	1 (10.0%)	6 (31.6%)	7 (31.8%)
Re-sternotomy	2 (20.0%)	4 (21.1%)	4 (18.2%)
Duration of surgery (min)	425.90 ± 86.46	452.32 ± 108.00	531.67 ± 142.52
CPB-time (min)	242.20 ± 65.57	234.58 ± 54.90	247.55
Cross-clamp-time (min)	120.80 ± 38.67	112.74 ± 44.96	112.14 ± 36.39
Cerebral perfusion (min)	68.50 ± 37.30	70.50 ± 27.10	85.27 ± 27.25
Subsequent endovascular procedure (TEVAR/BEVAR)	3 (30.0%)	10 (52.6%)	12 (54.5%)
Survival 1 y 2 y 3 y	7 (70.0%) 6 (60.0%) 6 (60.0%)	13 (68.4%) 10 (52.6%) 8 (42.1%)	17 (77.3%) 15 (68.2%) 14 (63.6%)

Values shown as mean (± SD), median (IQR), or n (%). CPB: Cardiopulmonary Bypass; TEVAR: Thoracic Endovascular Aortic Repair; BEVAR: Branched Endovascular Aortic Repair.

**Table 6:** Outcomes by Prosthesis Generation.

Variable	E-vita Open (n = 23)	E-vita Open Plus (n = 11)	E-vita Open Neo (n = 17)
In-hospital mortality	0 (0.0%)	0 (0.0%)	4 (23.5%)
EuroSCORE II	4.82 (1.92–6.13)	3.56 (1.75–4.24)	3.32 (1.74–4.66)
Stroke	5 (21.7%)	0 (0.0%)	4 (23.5%)
Paraplegia	1 (4.3%)	1 (9.1%)	2 (11.8%)
Postoperative dialysis	5 (21.7%)	2 (18.2%)	7 (41.2%)
Re-sternotomy	3 (13.0%)	2 (18.2%)	5 (29.4%)
Duration of surgery (min)	538.00 ± 100.58	370.36 ± 71.43	476.94 ± 139.98
CPB-time (min)	265.57 ± 49.42	204.64 ± 37.73	233.29 ± 60.73
Cross-clamp-time (min)	130.52 ± 40.89	88.18 ± 33.04	108.53 ± 32.02
Cerebral perfusion (min)	94.43 ± 25.32	57.09 ± 28.46	67.38 ± 24.34
Subsequent endovascular procedure (TEVAR/BEVAR)	10 (43.5%)	8 (72.7%)	7 (41.2%)
Survival 1 y 2 y 3 y	18 (78.3%) 17 (73.9%) 15 (65.2%)	10 (90.9%) 10 (90.9%) 9 (81.8%)	9 (52.9%) 4 (23.5%) 4 (23.5%)

Values shown as mean (±SD), median (IQR), or n (%). CPB: Cardiopulmonary Bypass; TEVAR: Thoracic Endovascular Aortic Repair; BEVAR: Branched Endovascular Aortic Repair.

**Table 7:** Detailed Neurological Risk Factor Analysis.

Variable	Stroke Group (n = 9)	No Stroke Group (n = 42)
Age	61.44 ± 14.77	61.57 ± 9.60
Re-operation	6 (66.7%)	21 (50.0%)
Previous stroke	1 (11.1%)	4 (9.5%)
CPB-time (min)	244.67 ± 64.44	241.02 ± 54.79
Cross-clamp time (min)	112.11 ± 30.63	114.48 ± 41.52
Cerebral perfusion (min)	90.33 ± 19.91	73.74 ± 31.14
Circulatory arrest (min)	86.67 ± 35.30 (n = 3)	72.81 ± 33.88 (n = 27)
Perfusion strategy Unilateral Bilateral Trilateral	7 (77.8%) 1 (11.1%) 1 (11.1%)	29 (69.0%) 6 (14.3%) 7 (16.7%)
Prosthesis type Open Open Plus Open Neo	5 (55.6%) 0 (0.0%) 4 (44.4%)	18 (42.9%) 11 (26.2%) 13 (31.0%)
Max. Lactate (mmol/L)	8.5 (4.55–9.5)	6.75 (4.55–8.6)

Values shown as mean (± SD), median (IQR), or n (%). CPB: Cardiopulmonary Bypass.

**Table 8:** Detailed Renal Risk Factor Analysis.

Variable	Dialysis Group (n = 14)	No Dialysis Group (n = 37)
Age	64.21 ± 9.39	60.47 ± 11.0
Re-operation	12 (85.7%)	15 (40.5%)
Preoperative GFR < 60 (mL/min)	4 (28.6%)	4 (10.8%)
CPB-time (min)	251.00 ± 64.92	235.64 ± 51.16
Cross-clamp-time (min)	114.57 ± 49.34	113.00 ± 36.09
Cerebral perfusion (min)	80.25 ± 27.0	74.74 ± 30.98
Circulatory arrest (min)	68.43 ± 33.59 (n = 7)	75.96 ± 34.34 (n = 23)
RBCs (mL)	1650 (1425–3375) (n = 14)	1500 (712.5–2100) (n = 30)
Platelets (mL)	900 (600–2100) (n = 14)	900 (675–1225) (n = 29)
FFP (mL)	2600 (975–4500) n = 8)	1350 (900–2700) (n = 20)
PCC (I.U.)	6000 (4375–8000) (n = 14)	4750 (4000–6000) (n = 32)
Fibrinogen (g)	6 (4.5–10) (n = 13)	5 (4–8) (n = 32)
Stroke	5 (35.7%)	4 (10.8%)
Re-sternotomy	5 (35.7%)	5 (13.5%)
Max. Lactate (mmol/L)	6.7 (4.55–10.38)	6.75 (4.53–8.5)
LOS ICU (d)	21 (9.75–28)	5 (3–7.75)
Time to discharge (d)	35 (27.75–40.75)	18.5 (14.75–22.25)
In-hospital mortality	2 (14.3%)	2 (5.4%)

Values shown as mean (± SD), median (IQR), or n (%). GFR: Glomerular Filtration Rate; CPB: Cardiopulmonary Bypass; RBCs: Red Blood Cells; FFP: Fresh Frozen Plasma; PCC: Prothrombin Complex Concentrate; LOS: Length of Stay; ICU: Intensive Care Unit.

## Data Availability

The data underlying this article are available in the article.

## Supplementary Materials

The following supporting information can be downloaded at: https://www.mdpi.com/article/10.3390/jcdd12090368/s1, Video S1: Implantation of the E-vita Open Neo Prosthesis.

## Abbreviations

The following abbreviations are used in this manuscript:

ASCP	Antegrade Selective Cerebral Perfusion
BEVAR	Branched Endovascular Aortic Repair
COPD	Chronic Obstructive Pulmonary Disease
CPB	Cardiopulmonary Bypass
CS	Carotid-Subclavian
CSF	Cerebrospinal Fluid
CT	Computed Tomography
FET	Frozen Elephant Trunk
FFP	Fresh Frozen Plasma
GFR	Glomerular Filtration Rate
ICU	Intensive Care Unit
IQR	Interquartile Range
MRI	Magnetic Resonance Imaging
NIRS	Near-Infrared Spectroscopy
OAD	Oral Antidiabetic Drugs
PCC	Prothrombin Complex Concentrate
RBCs	Red Blood Cells
SD	Standard Deviation
TAR	Total Arch Replacement
TEVAR	Thoracic Endovascular Aortic Repair
